# Potential of cell-free supernatants from cultures of selected lactic acid bacteria and yeast obtained from local fermented foods as inhibitors of *Listeria monocytogenes*, *Salmonella* spp. and *Staphylococcus aureus*

**DOI:** 10.1186/1756-0500-7-606

**Published:** 2014-09-04

**Authors:** Solomon H Mariam, Nigus Zegeye, Tewodros Tariku, Emawayish Andargie, Nigatu Endalafer, Abraham Aseffa

**Affiliations:** Aklilu Lemma Institute of Pathobiology, Addis Ababa University, Addis Ababa, Ethiopia; Armauer Hansen Research Institute, P.O. Box 1005, Addis Ababa, Ethiopia; Debre Berhan University, Debre Berhan, Ethiopia

**Keywords:** Lactic acid bacteria, Listeria monocytogenes, Salmonella, Staphylococcus aureus, Inhibition, Cell-free supernatant, Inhibition

## Abstract

**Background:**

Food-borne infections cause huge economic and human life losses worldwide. The most common contaminants of foods include *Listeria monocytogenes Salmonellae* and *Staphylococcus* aureus. *L. monocytogenes* is most notorious due to its tolerance to common food preservation methods and the risks it poses, including higher fatality rates. Safer, more efficacious control methods are thus needed. Along with food-borne pathogens, lactic acid bacteria (LAB) can also be found in foods. Some LAB isolates inhibit pathogenic bacteria by various mechanisms, including by production of antimicrobial metabolites.

**Methods:**

The potential of cell-free culture supernatants (CFS) derived from broth cultures of selected local LAB and yeast isolates, some of which were subjected to various treatments, were tested for inhibition of *L. monocytogenes*, *Salmonella* spp. and *S. aureus* in *in vitro* culture by incorporating various proportions of the CFSs into the growth medium concurrently with inoculation (co-cultures) or following limited proliferation after inoculation of the pathogens (delayed cultures). The effects of the CFSs on various growth parameters were assessed.

**Results:**

CFS from the LAB isolates were strongly inhibitory when co-cultured. The inhibitory activities were stable following heat or protease treatment of the CFSs. Inhibitory activity was dependent primarily on active substance(s) secreted into the supernatant. In all co-cultures, CFS proportion-dependent progressive decrease in the number of colonies was observed and both growth rates and number of generations were reduced with significantly fewer numbers of colony forming units, whereas generation times were significantly increased compared to those of controls. Transfer from co-cultures to fresh broth showed inhibited cultures contained bacteria that can re-grow, indicating the presence of viable bacteria that are undetectable by culture. Growth rates in CFS-treated delayed cultures were also reduced to varying degrees with the number of colonies in some cultures being significantly less than the corresponding control values. CFSs were active against both Gram-positive and –negative bacteria.

**Conclusions:**

Active metabolites produced and secreted by LAB into the growth medium were effective in inhibiting the tested pathogens. Early addition of the CFSs was necessary for significant inhibition to occur. Further studies will help make these findings applicable to food safety.

## Background

Food-borne infections cause huge economic losses, in addition to morbidity and mortality. *Listeria monocytogenes* and nontyphoidal *Salmonella enterica* ranked among the top five identifiable pathogens causing these human and economic losses [1]. A study estimated an aggregate annual cost of 50–77 billion due to food-borne illness in the United States [2]. *Staphlococcus aureus* also ranked among the top five pathogens that caused food-borne illnesses in 2011 [3].

*L. monocytogenes* is exceptional in that it can tolerate high salt concentrations, remains viable at temperatures as low as 0°C, and is also difficult to eliminate by ordinary disinfectants [4, 5]. It can be found as a contaminant in meat, milk and other food-processing facilities. Risk groups in humans include immuno-compromised persons and the elderly. The infection causes septicemia, meningitis, encephalitis or abortion or stillbirth of neonates, while gastroenteritis can occur in the general public [6]. The infectious dose is unknown but the case fatality rate has been reported to be 20-40% [6, 7], which is much higher than the fatality rate of salmonellosis, the incidence rate of which is, in turn, much higher than listeriosis [6, 7].

As well as food-borne pathogens, lactic acid bacteria (LAB) can also be found in various habitats including in foods and drinks of plant and animal origins. Fermented foods and drinks are particularly rich in their content of LAB. LAB species of practical importance are usually nonpathogenic and safe, at least in healthy and immuno-competent people. Several reports have indicated that the shelf-life of foods can be extended by LAB or LAB metabolites [8–11]. Food safety is enhanced because of the inhibitory actions of LAB on common food-borne pathogens such as *Escherichia coli* O157::H7, *Salmonella* spp. and *Listeria monocytogenes*. Inhibition of food-borne pathogens by LAB can be mediated by competitive exclusion or production of organic acids and antimicrobial products such as hydrogen peroxide and antimicrobial peptides (e.g., bacteriocins) [12–14]. These products can be produced by some LAB and secreted when grown in appropriate broth medium. Either live LAB or their metabolic products can be used [14]. Moreover, the antimicrobial properties of some LAB or their antimicrobial peptide products as well as their vaccine potential may make them suitable candidates as bio-therapeutic agents for human and animal applications [15–20]. Thus, selected LAB species with these and additional qualities (termed probiotics, defined as live microorganisms which, when administered in adequate amounts confer health benefit on the host [21]) have been developed.

In this work, we focused on the inhibitory effects of metabolites of a collection of LAB and yeast isolates obtained from local fermented foods and milk on some common food-borne pathogens or standard strains. The choice of these fermented foods and milk is based on the fact that these are consumed regularly by the population. The inhibitory effect of cell-free culture supernatants (CFS) from broth cultures of the selected isolates on growth rates and colony-forming units of *L. monocytogenes*, *Salmonella* and *S. aureus* were tested following various treatments of the CFS as described below.

## Methods

### Bacterial isolates and growth conditions

The sources of LAB used in this study were fermented milk, ananas and a grain called Teff, which are important fermented foods consumed regularly in Ethiopia. The fermentates were diluited in sterile PBS buffer, pH 7.2 and, to obtain single colonies, 100 μL of the sixth and seventh 10-fold serial dilutions were plated on De Man, Rogosa, Sharpe (MRS) agar, followed by picking single colonies at random and growing in MRS broth. Incubations of both agar and broth cultures were at 37°C at aerobic or anaerobic conditions. Combinations of standard tests (microscopy, cultural and biochemical) [22, 23] were used to tentatively identify the isolates. Cell pellets from the MRS broth cultures were resuspended in MRS broth containing 15% glycerol and aliquots were frozen for use when needed. All media used here were from OXOID, UK. In addition, while we were searching for a LAB isolate from the ananas fermentate, a non-LAB isolate that was found and later identified to be a yeast was used in the inhibition studies.

The bacteria tested for inhibition by the CFSs (target strains) were *Listeria monocytogenes* (ATCC 19115), *Salmonella enterica* subsp. enterica serovar Enteritidis (ATCC 13076) and *S. aureus* (ATCC 25923), all type strains. Another bacterial isolate used was MI1, an isolate from bovine milk identified to be *Salmonella* spp. by tests including Gram staining, colonial morphology, motility and growth on differentiating and selective media (MacKonkey, XLD and *Salmonella*-*Shigella* agars), sugar fermentation test with Biomerioux API20 (Biomerioux, France) in comparison with the known properties of *Salmonella* spp. The results of none of these tests conducted on the milk isolate were similar to those of the known properties of the possible contaminants of milk, such as *S. aureus*, *Listeria*, *E. coli*, *Brucella*, *Campylobacter* or *Mycobacterium* spp. [22, 23].

*L. monocytogenes* was grown in tryptic soy broth (TSB) or tryptic soy agar (TSA). *Salmonellae* and *S. aureus* were grown in Luria Bertani (LB) broth or agar. When applicable, agar media were supplemented with 0.5% pyruvate to enhance detection of possible presence of viable but nonculturable organisms.

### Growth of strains for preparation of CFS

Seventeen isolates from the above sources were grown in MRS broth as described above. The supernatant was recovered by centrifugation at 3000 rpm in a Beckman Allegra 6R (Beckman Coulter) centrifuge for 1 hr. CFSs were obtained by passing the supernatants through 0.4 μm pore size filters and tested for inhibitory effect on the tested bacteria (in co-cultures, please see below) before adjusting the pH of the CFSs. Four isolates (total LAB and *Lactococcus* sp. [both isolated from fermented milk], *Streptococcus thermophilus* [isolated from Teff] and a yeast species [isolated from ananas]) whose CFSs inhibited the bacteria when tested individually were selected based on this initial observation. These four isolates were grown individually from frozen stock samples in 20 mL MRS broth and incubating aerobically or anaerobically at 37°C. In addition, a cocktail made of a mixed culture of these four isolates (Labmix) was also grown in the same way as for the individual isolates. After 18 h of incubation, an aliquot of 100 μL was transferred into 250 mL of MRS broth and incubated for 24 h. After centrifugation as above, each supernatant was then divided into two portions and subjected successively to the following treatments, further dividing each supernatant into two in going from one treatment to the next.

### pH adjustment

The pH of the supernatants before pH adjustment were in the range of 4.5-4.8. A portion of each supernatant was subject to pH adjustment to 6.5 - 6.8.

### Proteinase K treatment of supernatant

Portions of supernatants were treated with proteinase K (BDH Biochemicals) at a concentration of 50 μg mL^-1^ for 1 hr at 37°C in a reaction mixture containing 0.5% SDS, 0.01 M Tris and 0.005 M EDTA [24]. Following treatment, the enzyme was inactivated by addition of phenylmethylsulfonyl fluoride. The supernatants were filtered through 0.4 μm pore size filter.

### Heat Treatment of CFS

To test if inhibitory activity was heat-stable or –sensitive, portions were heat-treated (100°C for 1 hr).

Thus, for each isolate, four types of CFS derived from it were tested for inhibitory activity: both pH-adjusted and heat-treated (CFS1), pH-adjusted only (CFS2), heat-treated only (CFS3), and neither pH-adjusted nor heat treated (CFS4). CFS prepared as above was aliquoted and stored at -20°C. A fresh aliquot was thawed and used for experiments.

In addition to MRS broth, LAPTg broth was also used to grow the LAB isolates and obtain the CFS. Catalase treatment of the CFSs from LAPTg broth was also conducted to test for possible involvement of hydrogen peroxide in inhibition which, if present, would be abolished by the catalase tretament.

### Test of CFS inhibitory effect

The CFSs were initially tested on the test bacteria *L. monocytogenes*, *Salmonellae* and *S. aureus* by adding CFS to 12.5%, 25% or 50% proportions (v/v) in 5 mL final volume. Intermediate CFS proportions were also included as deemed necessary. In either case, 1 μL each of *L. monocytogenes* (~8.00 × 10^3^ CFU mL^-1^), *S. enterica* (~2.60 × 10^5^ CFU mL^-1^), *Salmonella* (MI1) (~1.32 × 10^4^ CFU mL^-1^) or *S. aureus* (~1.30 × 10^4^ CFU mL^-1^) from frozen stocks were inoculated. The CFSs were added in two different modes to make the 5 mL final volume according to their percentage proportions: (i) Co-cultures, in which CFS were added simultaneously with inoculation of the test bacteria; (ii) Delayed cultures, in which broth media were inoculated and incubated, followed by CFS addition after 6 hrs of incubation. To obtain the rate of growth of the target strains the turbidity was measured using an optical density of 600 nm with a spectrophotometer Novaspec II (Pharmacia Biotech) as well as by plating 1 μL of 10-fold serial dilutions of (i) the co-cultures at time points day 0 (immediately after both inoculation and CFS addition) and 3 days and (ii) the delayed cultures at time points day 0 (immediately after CFS addition, i.e., after 6 hrs of inoculation and incubation) and 1.5 days. Growth controls for co-cultures included cultures in 5 mL broth volumes without added CFS. Cultures in which MRS broth was added instead of CFS (to 50% v/v) were also included. Colonies were usually enumerated after 24 or 48 hrs on TSA agar (*L. monocytogenes*) or LB agar (*Salmonellae and S.aureus*).

When no growth was obtained, a sample was withdrawn and inoculated into fresh TSB or LB broth without added CFS and growth monitored as for the CFS-treated cultures. In addition, appearance of colonies was assessed by plating 10 or 50 μL samples to see if there were surviving cells that were detectable by culture.

### Determination of growth rates, generation times, and number of generations

CFS that showed strong inhibition was selected for each test bacterium and added at the lower inhibitory proportion (30% for *L. monocytogenes*, 20% for *S.aureus* and 20% for *Salmonella* MI1, and *S. enterica*). Then, rate of growth was quantified by both turbidity measurement and plating on agar media at two hr intervals starting from one hr after incubation of inoculated cultures and continuing during the exponential growth phase. The generation times (G) were calculated from the relationship *G* = ln2/*k*, where,


with N_1_ being the number of bacteria at time T_1_ (endpoint) and N_0_ being the number of bacteria at time T_0_ (start of logarithmic growth phase). Number of generations (N) was obtained from:


Each experiment was conducted three independent times and data represent the averages of these experiments. The Wilcoxon Rank Sum test was used to determine if significant differences exist between means of bacterial numbers (CFUs) of control and experimental cultures.

### Test of MRS effect

To test if any of the components of the pure MRS broth itself could have role in the inhibition, fresh MRS broth was added to the TSB or LB broth to 50% (v/v) proportion (in co-cultures) and inoculated with the target bacteria and the growth compared to those of the controls (i.e., those grown in 100% TSB or LB).

## Results

The pH of the CFSs before pH adjustment were in the range of 4.5-4.8. Only CFS3 and CFS4 obtained from the four selected isolates and the Labmix exhibited inhibitory activity; i.e., pH-adjusted CFSs were ineffective in inhibition. Inhibitory effect was not abolished by treatment with the protease. The selected isolates were tentatively identified as total LAB and *Lactococcus* sp. (both isolated from fermented milk), *Streptococcus thermophilus* (isolated from Teff) and a yeast species isolated from ananas.

### Effect of CFS on growth rates and generation times

In the presence of CFS, both growth rates and number of generations were significantly reduced when compared to those of the controls. Thus, the growth rates of *L. monocytogenes*, MI1 and *S. aureus* experimental cultures were reduced by 40%, 38% and 32% respectively in comparison to those of their respective controls. Accordingly, the number of generations of the experimental cultures decreased by the same percentage points as did the growth rates (P < 0.05). Conversely, the generation times of experimental cultures of the three bacteria were significantly increased compared to their respective controls (by 164%, 162% and 147% respectively). Overall, MI1 (the milk isolate) had the highest growth rate, the shortest generation time and the highest number of generations among the three bacteria in both the control and experimental conditions (Table 1).

**Table 1:** **Growth rates and generation times and numbers of bacteria in control (CFS-free) and experimental (CFS-treated) cultures**

Bacteria	Growth rate ( ***k***, hr ^-1^)		Generation time (minutes)		No of generations	
	Control	Exp.	Control	Exp.	Control	Exp.
*L. monocytogenes*	1.9 ± 0.002	1.15 ± 0.006	21.8 ± 0.025	35.9 ± 0.004	10.97 ± 0.013	6.65 ± 0.03
*Salmonella* (MI1)	2.9 ± 0.03	1.8 ± 0.015	14.3 ± 0.14	23.2 ± 0.20	16.73 ± 0.17	9.83 ± 0.59
*S. aureus*	1.64 ± 0.008	1.12 ± 0.09	25.4 ± 0.13	37.4 ± 3.19	9.46 ± 0.033	6.45 ± 0.55

(Exp. = Experimental).

### Simultaneous cultures

Plating 1 μL of dilutions of the *L. monocytogenes* co-cultures on day 3 from the 50% CFS proportions of total LAB, *Lactococcus* and Labmix gave no colonies, but after plating 50 μL, 2.26 × 10^2^ CFU mL^-1^ was obtained from the *Lactococcus* CFS co-culture only. However, on day 3, the average CFU from the control cultures was 1.71 × 10^9^ ± 4.77 × 10^8^, an increase of 4.72 Log units over that of day 0 control CFU. CFS from *S. thermophiles* and yeast were less effective against *L. monocytogenes* but nonetheless, the CFU obtained were significantly less than the control (P ≤ 0.05) (Table 2A).

**Table 2:** **Log CFU mL** ^**-1**^ **of CFS-treated** ***L. monocytogenes*** **(A) and** ***S. enterica*** **(B) co-cultures**

A. *L. monocytogenes*						
Day	CFS (%)		CFS			
		Total LAB	*Lactococcus*	Labmix	*S. thermophilus*	Yeast sp.
0	0		4.00 × 10^4^ ± 2.64 × 10^3^			
3	0		1.71 × 10^9^ ± 4.77 × 10^8^			
	25	1.90 × 10^8^ ± 1.13 × 10^8⊕^	8.00 × 10^7^ ± 2.83 × 10^7^	1.50 × 10^8^ ± 1.41 × 10^7^	3.60 × 10^7^ ± 1.41 × 10^6^	1.82 × 10^7^
	50	0.0^Ω^	2.26 × 10^2^ ± 3.05 × 10^1♦^	0.0^Ω^	6.25 × 10^4^ ± 1.06 × 10^4^	6.80 × 10^7^ ± 2.83 × 10^6^
B. *S. enterica*						
Day	CFS (%)		CFS			
		Total LAB	*Lactococcus*	Labmix	*S. thermophilus*	Yeast sp.
0	0		1.30 × 10^6^ ± 1.00 × 10^5^			
3	0		1.83 × 10^8^ ± 1.15 × 10^7^			
	12.5	2.50 × 10^2^ ± 7.0 × 10^1^	5.00 × 10^4^ ± 0.0^∑^	4.20 × 10^3^ ± 4.24 × 10^2^	8.40 × 10^3^ ± 5.65 × 10^2^	ND

Comparisons were made between CFUs from all CFS-treated co-cultures on day 3 and CFU from the control **(**0% CFS**)** on day 3. CFUs from the day 0 control (0% CFS) co-cultures are for reference only, i.e., to indicate both initial CFUs and that growth occurs in the absence of CFS treatment.

^⊕^For *L. monocytogenes*, this value was significantly less than its respective day 3 control CFU (P <0.05). Since all other experimental CFUs of all CFSs on day 3 were less than this value, they were also significantly less than the day 3 control CFU.

♦ - Obtained by plating 50 μL (undiluted) instead of the usual 1 μL of 10^-6^ or 10^-4^ dilutions.

Ω - This value was zero even after plating 50 μL direct from culture instead of 1 μL of dilutions.

^∑^For *S. enterica*, this value was significantly less than its respective day 3 control CFU (P <0.05). Since all other experimental CFUs of all CFSs on day 3 were less than this value, they were also significantly less than the day 3 control CFU.

ND – Not done.

The *S. enterica* co-cultures exhibited the most dramatic reductions in CFU by day 3, and this was obtained with the lowest CFS proportions (12.5%). The 12.5% proportions of all CFSs caused a > 99% reduction in CFU compared to the day 3 control CFUs, while the latter increased by > 140 fold over that of day 0 CFU (i.e., an increase of 2.15 Log units (Table 2B).

The control co-culture of MI1 continued to grow into day 3 of culture by an increase of 4.47 Log units relative to that of day 0 control (2.06 × 10^9^ ± 1.58 × 10^8^ vs 7.05 × 10^4^ ± 2.64 × 10^3^). Among the 12.5% CFS-treated cultures, Labmix and *S. thermophilus* caused the highest reductions in CFU, by 4.83 and 3.83 Log CFU mL^-1^ units respectively, relative to the day 3 control (*P* ≤ 0.05). With the highest CFS proportions tested (25%), the CFU were reduced to zero or to below the level of the day zero CFUs except for that of the yeast CFS (Table 3A). Since zero CFU were obtained for some of the samples with 25% CFS proportions, increased volumes (10 or 50 μL instead of the usual 1 μL) were subsequently plated on day 5 for those cultures which gave zero CFU on day 3. The result was that either the colonies so obtained were extremely small compared to those from the control or still no CFU were obtained.

**Table 3:** **Log CFU mL** ^**-1**^ **of CFS-treated** ***Salmonella*** **(MI1) (A) and** ***S. aureus*** **(B) co-cultures**

A. *Salmonella* MI1						
Day	CFS (%)		CFS			
		Total LAB	*Lactococcus*	Labmix	*S. thermophilus*	Yeast sp.
0	0		7.05 × 10^4^ ± 2.64 × 10^3^			
3	0		2.06 × 10^9^ ± 1.58 × 10^8^			
	12.5	1.90 × 10^7^ ± 1.41 × 10^6Δ^	9.90 × 10^7^ ± 1.41 × 10^6^	3.06 × 10^4^ ± 8.48 × 10^2*^	3.05 × 10^5^ ± 7.07 × 10^3^	2.10 × 10^8^ ± 2.82 × 10^7∞^
	25	0.0	3.20 × 10^4*^	0.0**	3.70 × 10^2^ ± 9.8 × 10^1^	2.51 × 10^7^ ± 1.41 × 10^5^
B. *S. aureus*						
Day	CFS (%)		CFS			
C.		Total LAB	*Lactococcus*	Labmix	*S. thermophilus*	Yeast sp.
0	0		6.27 × 10^4^ ± 6.43 × 10^3^			
3	0		1.05 × 10^9^ ± 2.12 × 10^8^			
	12.5	9.30 × 10^6^ ± 1.27 × 10^6^	6.00 × 10^7^ ± 4.24 × 10^7θ^	5.15 × 10^5^ ± 2.12 × 10^4^	1.95 × 10^7^ ± 1.41 × 10^6^	5.40 × 10^7^ ± 2.82 × 10^6^
	25	6.80 × 10^4^ ± 1.69 × 10^4^	8.50 × 10^5^ ± 4.94 × 10^5^	1.09 × 10^5^ ± 7.07 × 10^3^	0.0	2.37 × 10^7^ ± 2.40 × 10^6^

Comparisons were made between CFUs from all CFS-treated co-cultures on day 3 and CFU from the control on day 3. CFUs from the day 0 control (0% CFS) co-cultures are for reference only, i.e., to indicate both initial CFUs and that growth occurs in the absence of CFS treatment.

Δ - Colonies were extremely small even after prolonged incubation for 48 hrs.

* - Obtained by plating of 50 μL (undiluted) instead of the previous 1 μL (undiluted) plating.

** - This value was 0.0 even after plating 10 or 50 μL directly (without dilution) instead of 1 μL.

^∞^For *Salmonella* (MI1), this value was significantly less than its respective day 3 control CFU (P ≤ 0.05). Since all other experimental CFUs of all CFSs on day 3 were less than this value, they were also significantly less than the day 3 CFU.

^θ^For *S. aureus*, this value was significantly less than its respective day 3 control CFU (P ≤ 0.05). Since all other experimental CFUs of all CFSs on day 3 were less than this value, they were also significantly less than the day 3 CFU.

Increased reductions in CFU with increased CFS proportions were also seen with *S. aureus* co-cultures, exhibiting the highest reductions at the 25% CFS, but even the 12.5% CFS also caused significant reductions in CFU at day 3 relative to that of the day 3 control (P ≤ 0.05) (Table 3B). Comparing CFUs obtained from corresponding CFS proportions, the 12.5% and 25% total LAB CFSs resulted in 2.05 and 4.19 Log decreases respectively on day 3 relative to the day 3 control; while that of the control increased by 4.21 Log units relative to the initial, day 0 control. Similarly, the 12.5 and 25% CFS of *Lactococcus* caused 1.24 and 3.09 Log units decrease relative the day 3 control CFU.

### Delayed cultures

The *L. monocytogenes* delayed culture was more resistant to the CFSs than the other bacteria. However, it was kept to at least below the control CFU level by day 1.5 and most cultures gave CFU that were significantly less than the control (*P* ≤ 0.05) (Table 4A). There was no CFS proportion-dependent progressive decrease in CFU of *L. monocytogenes* delayed cultures.

**Table 4:** **Log CFU mL** ^**-1**^ **of CFS-treated** ***L. monocytogenes*** **(A),** ***S. aureus*** **(B) and** ***Salmonella*** **(MI1) (C) delayed cultures**

A. *L. monocytogenes*					
Day	CFS (%)		CFS		
		Total LAB	*Lactococcus*	Labmix	*S. thermophilus*
0	0		1.38 × 10^6^ ± 2.56 × 10^4^		
	30		1.45 × 10^6^ ± 1.06 × 10^4^		
	40		1.88 × 10^6^ ± 1.52 × 10^4^		
	50		1.68 × 10^6^ ± 2.82 × 10^4^		
1.5	0		1.28 × 10^9^ ± 7.03 × 10^8^		
	30	3.35 × 10^8^ ± 3.53 × 10^7*^	8.50 × 10^8^ ± 7.07 × 10^7**^	2.05 × 10^8^ ± 7.07 × 10^6*^	4.40 × 10^8^ ± 8.48 × 10^7**^
	40	ND	1.55 × 10^8^ ± 3.53 × 10^7*^	4.15 × 10^8^ ± 7.77 × 10^7*^	5.80 × 10^8^ ± 2.82 × 10^7**^
	50	5.25 × 10^8^ ± 7.07 × 10^6**^	8.50 × 10^8^ ± 7.07 × 10^6**^	2.90 × 10^8^ ± 1.41 × 10^7*^	2.55 × 10^8^ ± 6.36 × 10^7*^
B. *S. aureus*					
Day	CFS (%)		CFS		
		Total LAB	*Lactococcus*	Labmix	*S. thermophilus*
0	0		4.30 × 10^6^ ± 1.76 × 10^4^		
	20		3.57 × 10^6^ ± 3.53 × 10^4^		
	25		3.41 × 10^6^ ± 3.53 × 10^4^		
1.5	0		2.20 × 10^8^ ± 4.24 × 10^7^		
	20	8.50 × 10^7^ ± 7.07 × 10^6*^	8.60 × 10^8^ ± 8.48 × 10^7**^	2.85 × 10^8^ ± 2.12 × 10^7**^	2.15 × 10^8^ ± 7.07 × 10^6**^
	25	2.20 × 10^8^ ± 8.48 × 10^7**^	7.50 × 10^7^ ± 3.53 × 10^7*^	5.00 × 10^7^ ± 0.0^*^	9.00 × 10^7^ ± 2.82 × 10^7*^
C. *Salmonella* MI1					
Day	CFS (%)		CFS		
		Total LAB	*Lactococcus*	Labmix	*S. thermophilus*
0	0		1.55 × 10^6^ ± 4.24 × 10^4^		
	20		1.75 × 10^6^ ± 1.06 × 10^4^		
	25		1.56 × 10^6^ ± 2.82 × 10^4^		
1.5	0		5.90 × 10^8^ ± 3.39 × 10^7^		
	20	2.00 × 10^7^ ± 1.41 × 10^7*^	5.00 × 10^7^ ± 1.41 × 10^7*^	2.00 × 10^7^ ± 1.41 × 10^7*^	2.50 × 10^7^ ± 7.07 × 10^6*^
	25	ND	4.50 × 10^7^ ± 2.12 × 10^7*^	2.00 × 10^7^ ± 1.41 × 10^7*^	2.50 × 10^7^ ± 7.07 × 10^6*^

For each bacterium, those experimental CFUs that are significantly less than the respective day 1.5 control CFU are marked with (^*^), while those that are not are marked with (^**^). ND- not done.

The *S. aureus* delayed cultures were insensitive to the tested CFSs at 20%. However, all CFSs at 25% reduced the CFU significantly, except that of total LAB (*P* ≤ 0.05) (Table 4B).

With the *Salmonella* MI1 delayed cultures, day 1.5 CFU from treated cultures were less than the day 1.5 control by 1.07 – 1.47 Log units. (Table 4C), i.e., all day 1.5 experimental CFUs were significantly less than the day 1.5 control (*P* ≤ 0.05).

There was no CFS inhibitory effect on growth of *S. enterica* delayed cultures as there was no difference at all in CFUs between those grown in LB broth with or without any of the CFSs.

### MRS effect

For all target bacteria, there was no difference in the number of CFU obtained in the co-cultures containing 50% MRS broth (i.e., 50% MRS + 50% TSB or LB) compared to the controls (i.e., cultures in 100% TSB or LB).

## Discussion

In this work, CFSs derived from selected LAB and yeast broth cultures were tested for inhibitory effect on various bacteria in cultures to which CFS were added in simultaneous or delayed mode. Inhibitory effect was much stronger in co-cultures. All test bacteria were inhibited at all CFS proportions tested in co-cultures, with stronger inhibition at the higher CFS proportions. The inhibition of *S. enterica* in co-culture is particularly noteworthy for two reasons: the initial (day 0) CFU was the highest among those of all the target bacteria and the inhibition was exerted by the lowest CFs proportion.

The delayed cultures showed that even with late addition of CFS, it was possible to retard the growth, in most cases, to at least below the control level at the end of the experiment.

The inhibitory action of the CFSs was not abolished by treatments with proteinase K. The inhibitory substance(s) in the CFSs were heat-resistant as activity was not abolished after heat treatment. They do not appear to be of the class III non-bacteriocin-like lytic proteins [25, 26] due to their heat resistance. They were also equally active against both Gram-positive and –negative bacteria. Bacteriocin-like, heat-stable substances active against either or both groups of bacteria have been described [27–30]. These results suggest the non-protein (i.e., non-bacteriocin) nature of the active substances within the CFSs. Treatment with catalase did not result in abolition of activity either, which might indicate hydrogen peroxide was not responsible for the observed activities. There have been some reports about the unsuitability of MRS broth for growth and production of LAB CFS to be used in inhibition of certain pathogens due to its inhibition of production of hydrogen peroxide [31, 32]. The recommended medium instead was LAPTg broth. Accordingly, this broth was used here to grow LAB and obtain the CFS. The CFS so obtained did not result in enhanced inhibition of *L. monocytogenes* or *Salmonellae* or *S. aureus*. In fact, growth of these bacteria in CFS obtained from LAPTg broth-grown LAB was equal to or slightly higher than CFS from MRS broth-grown LAB. These results may be suggestive of a minor, if any, role for H_2_O_2_ in the observed inhibition in MRS broth CFS. Catalase treatment of the CFS obtained from the LAPTg broth culture did not result in enhanced growth of the target bacteria either. In addition, H_2_O_2_ in the CFSs would have had little, if any, role in inhibition, at least in the cases of *L. monocytogenes* and *S. aureus*, as these organisms are catalase producers themselves. Both of these findings (the non-protein and non-H_2_O_2_ nature of the active substances) bear an interesting similarity to the results of Bleicher et al. [33]. The inhibition observed in this work is not explainable as having been mediated by low pH alone as most of the 17 CFSs tested before pH adjustment were not effective. This ascribes the inhibitory effect largely, if not totally, to non-bacteriocin metabolites secreted by the LAB isolates into the growth medium. It is possible that there was a combined synergistic effect between low pH and the metabolites. Biochemical characterization of the substances is needed to ascertain their exact nature.

The effects of the CFSs in inhibiting the bacterial pathogens were transitory, as was also seen in the work of others [34, 35]. Colonies from experimental cultures were both fewer in numbers and much smaller in size. Upon transfer of samples from apparently completely inhibited cultures to fresh broth medium, however, growth occurred. After a second serial transfer, the resulting colonies attained comparable sizes to those from control cultures, indicating some bacterial populations remained viable but not culture-detectable.

## Conclusions

Pathogens such as *L. monocytogenes* with tolerance or resistance to common food preservation methods and disinfectants, exceptional adaptation to changing environments and persistence in food-processing facilities continue to pose risk to human health. The antibiotic arsenal is limited, and bacterial pathogens often develop resistance to newly introduced antibiotics. These facts dictate that, along with new antibiotics, suitable pathogen control alternatives that are safe and efficacious are needed. LAB are suitable candidates in the search for these alternatives, and globally, some are already used in probiotic formulations. In this work, it was demonstrably possible to significantly retard the growth of the tested bacteria by LAB metabolites. To the best of our knowledge, we are not aware of reports of studies on local fermented foods that simultaneously identified the LAB species and pathogenic bacteria within the specific fermented foods and studied the interactions between the two groups of bacteria. Similar other studies have been done elsewhere, but they do not necessarily apply universally. Even members of the same bacterial species can have contrasting virulence characters (an example would be the *Enterococci*: *Enterococcus faecalis*, a probiotic strain and a pathogenic clinical isolate of *Enterococcus faecalis*).

This study was limited in scope, but we intend to further this work by focusing not only on the antimicrobial properties of the LAB, but also isolation and identification of the metabolites, their mechanisms of action, suitable modes of application, other potential applications, safety issues including the presence of virulence factors and transferable antibiotic resistance and comparison to standard type strains. In addition, a medium-to-long term goal would be to promote and advance probiotic science in our setting, which is virtually unknown at this time. For this, full characterization of the resident microbial flora within fermented foods and drinks of local origin is an imperative.

## Abbreviations

LAB: Lactic acid bacteria
CFS: Cell-free supernatant
MRS: Mann, Rogossa, Sharpe
CFU: Colony-forming units
TSB: Tryptic soy broth
LB: Luria-Bertani.

## References

[1] Hoffmann S, Batz MB, Morris JG (2012). Annual cost of illness and quality-adjusted life year losses in the United States due to 14 foodborne pathogens. J Food Prot.

[2] Scharff RL (2012). Economic burden from health losses due to foodborne illness in the United States. J Food Prot.

[3] Food Safety: **Centers for disease control and prevention.** http://www.cdc.gov/foodsafety/facts.html

[4] Pan Y, Breidt F, Kathariou S (2006). Resistance of *Listeria monocytogenes* biofilms to sanitizing agents in a simulated food processing environment. Appl Environ Microbiol.

[5] Müller A, Rychli K, Muhterem-Uyar M, Zaiser A, Stessl B, Guinane CM, Cotter PD, Wagner M, Schmitz-Esser S (2013). Tn*6188* - a novel transposon in *Listeria monocytogenes* responsible for tolerance to benzalkonium chloride. PLoS One.

[6] Vázquez-Boland JA, Kuhn M, Berche P, Chakraborty T, Domínguez-Bernal G, Goebel W, González-Zorn B, Wehland J, Kreft J (2001). Listeria pathogenesis and molecular virulence determinants. Clin Microbiol Rev.

[7] Camejo A, Carvalho F, Reis O, Elsa Leitão E, Sousa S, Cabanes D (2011). The arsenal of virulence factors deployed by *Listeria monocytogenes* to promote its cell infection cycle. Virulence.

[8] Abee T, Krockel L, Hill C (1995). Bacteriocins: modes of action and potentials in food preservation and control of food poisoning. Int J Food Microbiol.

[9] Caplicec E, Fitzgerald GF (1999). Food fermentations: role of microorganisms in food production and preservation. Int J Food Microbiol.

[10] Natrajan N, Sheldon BW (2000). Inhibition of *Salmonella* on poultry skin using protein- and polysaccharide-based films containing a nisin formulation. J Food Prot.

[11] Benkerroum N, Oubel H, Sandine WE (2003). Effect of nisin on yogurt starter, and on growth and survival of *Listeria monocytogenes* during fermentation and storage of yogurt. Int J Food Saf.

[12] De Vuyst L, Leroy F (2007). Bacteriocins from lactic acid bacteria: production, purification, and food applications. J Mol Microbiol Biotechnol.

[13] Atassi F, Servin AL (2010). Individual and co-operative roles of lactic acid and hydrogen peroxide in the killing activity of enteric strain Lactobacillus johnsonii NCC933 and vaginal strain Lactobacillus gasseri KS120.1 against enteric, uropathogenic and vaginosis-associated pathogens. FEMS Microbiol Lett.

[14] O’Shea EF, Cotter PD, Ross RP, Hill C (2013). Strategies to improve the bacteriocin protection provided by lactic acid bacteria. Curr Opin Biotechnol.

[15] Dabour N, Zihler A, Kheadr E, Lacroix C, Fliss I (2009). In vivo study on the effectiveness of pediocin PA-1 and Pediococcus acidilactici UL5 at inhibiting Listeria monocytogenes. Int J Food Microbiol.

[16] Riley MA, Robinson SM, Roy CM, Dennis M, Liu V, Dorit RL (2012). Resistance is futile: the bacteriocin model for addressing the antibiotic resistance challenge. Biochem Soc Trans.

[17] Goh Y-L, He H, March JC (2012). Engineering commensal bacteria for prophylaxis against infection. Curr Opin Biotechnol.

[18] Wells J (2011). Mucosal vaccination and therapy with genetically modified lactic acid bacteria. Ann Rev Food Sci Technol.

[19] Koo OK, Amalaradjou MAR, Bhunia AK (2012). Recombinant probiotic expressing Listeria adhesion protein attenuates Listeria monocytogenes virulence in vitro. PLoS One.

[20] Cotter PD, Ross RP, Hill C (2013). Bacteriocins - a viable alternative to antibiotics?. Nat Rev Microbiol.

[21] Joint Food and Agriculture Organization of the United Nations/World Health Organization: **Working Group Report on Drafting Guidelines for the Evaluation of Probiotics in Food, London, Ontario, Canada.** April 30 and May, 2002 [cited 2010 Aug 25]. ftp://ftp.fao.org/es/esn/food/wgreport2.pdf

[22] MacFadden RR (2000). Biochemical Tests for Identification of Medical Bacteria.

[23] Murray PR, Shea Y (2004). Guide to Clinical Microbiology.

[24] Sambrook J, Fritsch EF, Maniatis T (1989). Molecular Cloning: A Laboratory Manual.

[25] Cleveland J, Montville TJ, Nes IF, Chikindas ML (2001). Bacteriocins: safe, natural antimicrobials for food preservation. Int J Food Microbiol.

[26] Cotter PD, Hill C, Ross RP (2005). BACTERIOCINS: developing innate immunity for food. Nat Rev Microbiol.

[27] Jack RW, Tagg JR, Ray B (1995). Bacteriocins of gram-positive bacteria. Microbiol Rev.

[28] Mélançon D, Grenier D (2003). Production and properties of bacteriocin-like inhibitory substances from the swine pathogen Streptococcus suis serotype 2. Appl Environ Microbiol.

[29] Walls T, Power D, Tagg J (2003). Bacteriocin-like inhibitory substance (BLIS) production by the normal flora of the nasopharynx: potential to protect against otitis media?. J Med Microbiol.

[30] Gálvez A, Abriouel H, Benomar N, Lucas R (2010). Microbial antagonists to food-borne pathogens and biocontrol. Curr Opin Biotechnol.

[31] Rodríguez JM, Martínez MI, Suárez AM, Martínez JM, Hernández PE (1997). Research note: unsuitability of the MRS medium for the screening of hydrogen peroxide-producing lactic acid bacteria. Lett Appl Microbiol.

[32] Pridmore RD, Pittet AC, Praplan F, Cavadini C (2008). Hydrogen peroxide production by Lactobacillus johnsonii NCC 533 and its role in anti-Salmonella activity. FEMS Microbiol Lett.

[33] Bleicher A, Stark T, Hofmann T, Bogovic Matijasić B, Rogelj I, Scherer S, Neuhaus K (2010). Potent antilisterial cell-free supernatants produced by complex red-smear cheese microbial consortia. J Dairy Sci.

[34] Bouttefroy A, Millie’re J-B (2000). Nisin–curvaticin 13 combinations for avoiding the regrowth of bacteriocin resistant cells of *Listeria monocytogenes* ATCC 15313. Int J Food Microbiol.

[35] Hartmann HA, Wilke T, Erdmann R (2011). Efficacy of bacteriocin-containing cell-free culture supernatants from lactic acid bacteria to control *Listeria monocytogenes* in food. Int J Food Microbiol.

